# Supplementary figures and images for: Interplay of adherens junctions and matrix proteolysis determines the invasive pattern and growth of squamous cell carcinoma
Source: eLife. 2023 Mar 9;12:e76520. doi: 10.7554/eLife.76520 (PMC9998089; doi:10.7554/eLife.76520)

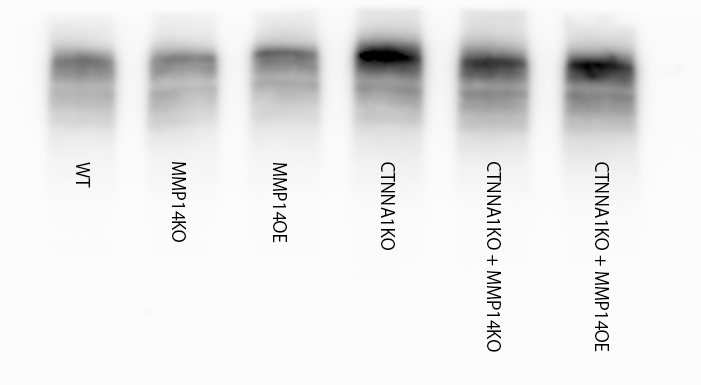

Supplement: Figure 3—figure supplement 1—source data 2. [file elife-76520-fig3-figsupp1-data2.zip › Figure 3 - figure supplement 1 - source data2/Figure 3 - figure supplement 1f Blot of Fibronectin with label.png]

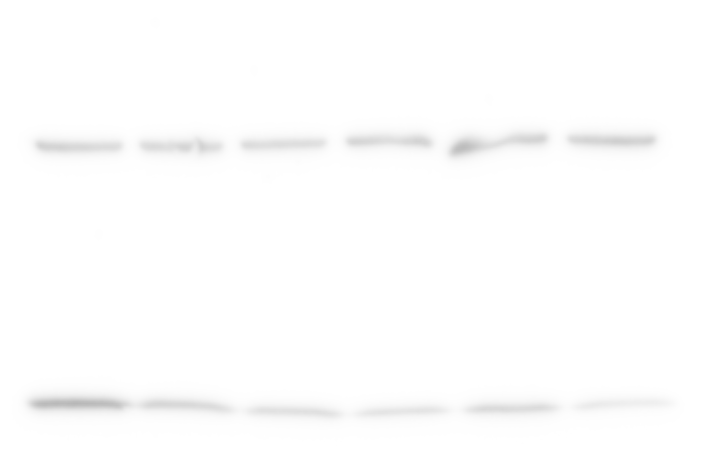

Supplement: Figure 3—figure supplement 1—source data 2. [file elife-76520-fig3-figsupp1-data2.zip › Figure 3 - figure supplement 1 - source data2/Figure 3 - figure supplement 1f Blot of beta actin.png]

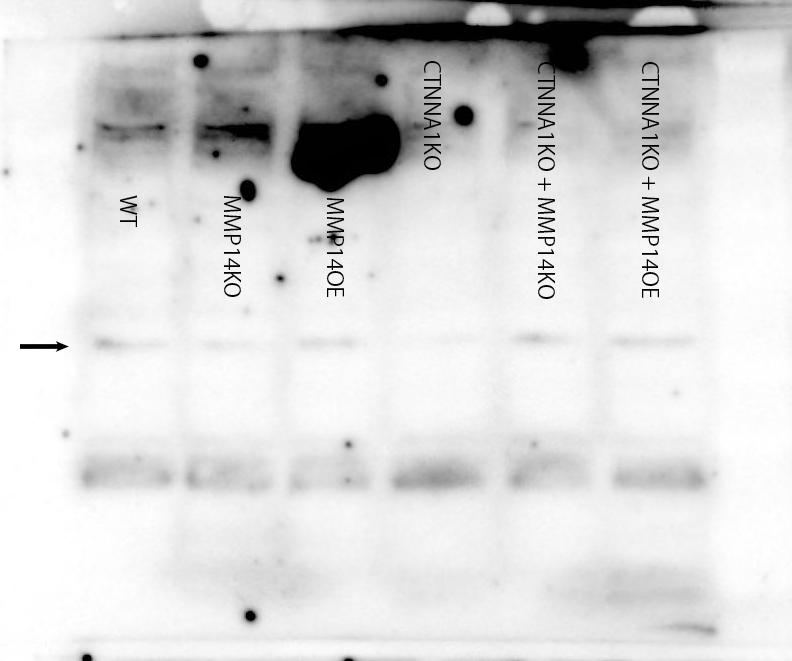

Supplement: Figure 3—figure supplement 1—source data 2. [file elife-76520-fig3-figsupp1-data2.zip › Figure 3 - figure supplement 1 - source data2/Figure 3 - figure supplement 1f Blot of Vimentin with label.png]

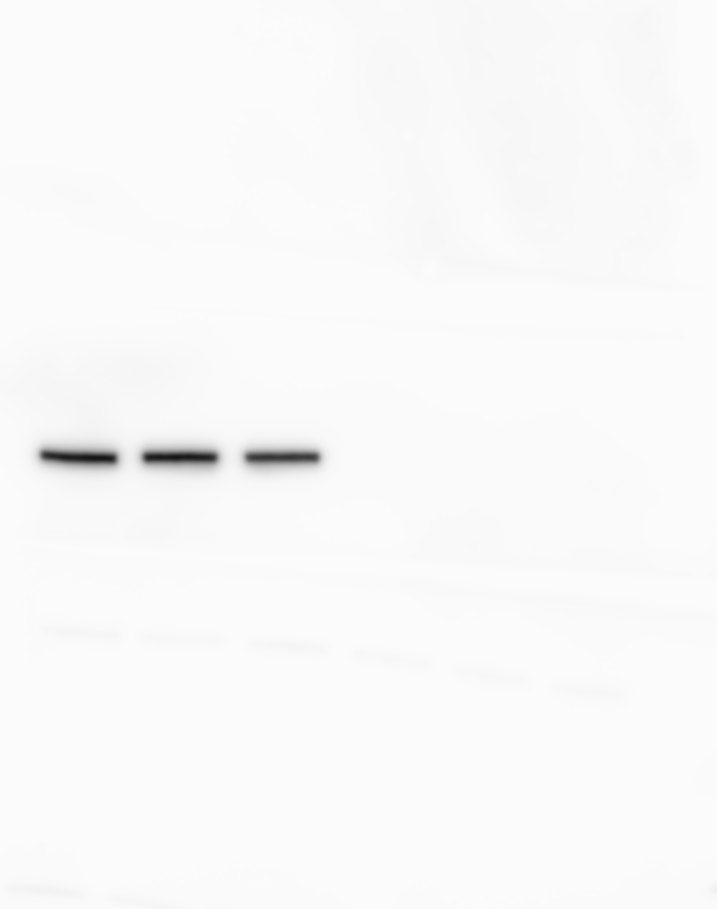

Supplement: Figure 3—figure supplement 1—source data 2. [file elife-76520-fig3-figsupp1-data2.zip › Figure 3 - figure supplement 1 - source data2/Figure 3 - figure supplement 1f Blot of alpha catenin.png]

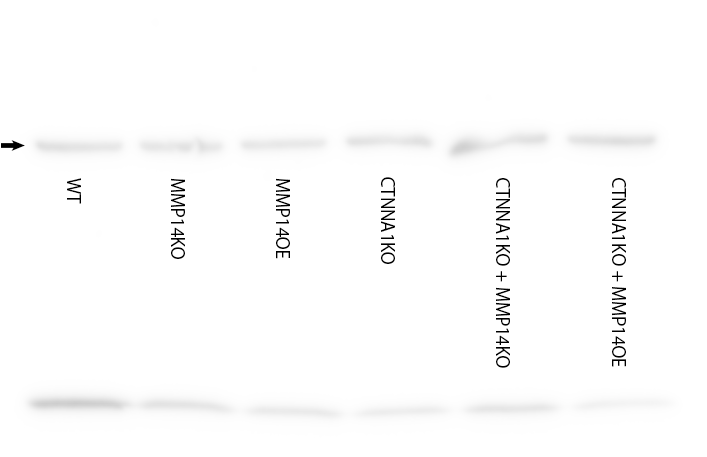

Supplement: Figure 3—figure supplement 1—source data 2. [file elife-76520-fig3-figsupp1-data2.zip › Figure 3 - figure supplement 1 - source data2/Figure 3 - figure supplement 1f Blot of beta actin with label.png]

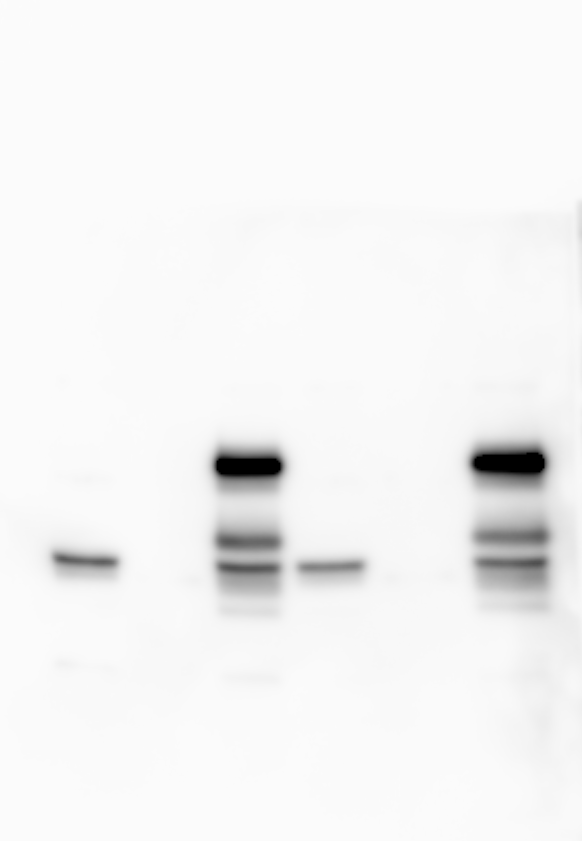

Supplement: Figure 3—figure supplement 1—source data 2. [file elife-76520-fig3-figsupp1-data2.zip › Figure 3 - figure supplement 1 - source data2/Figure 3 - figure supplement 1f Blot of MMP14.png]

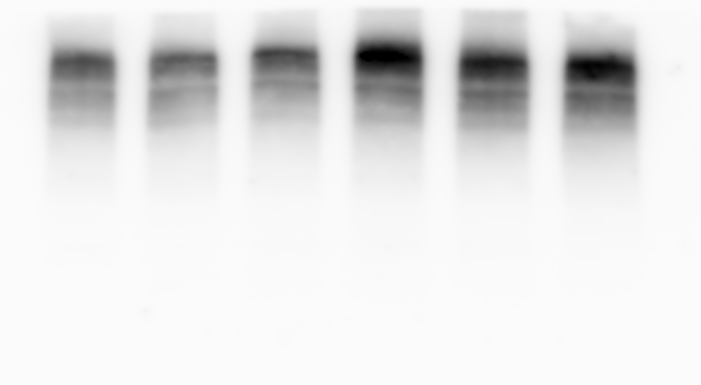

Supplement: Figure 3—figure supplement 1—source data 2. [file elife-76520-fig3-figsupp1-data2.zip › Figure 3 - figure supplement 1 - source data2/Figure 3 - figure supplement 1f Blot of Fibronectin.png]

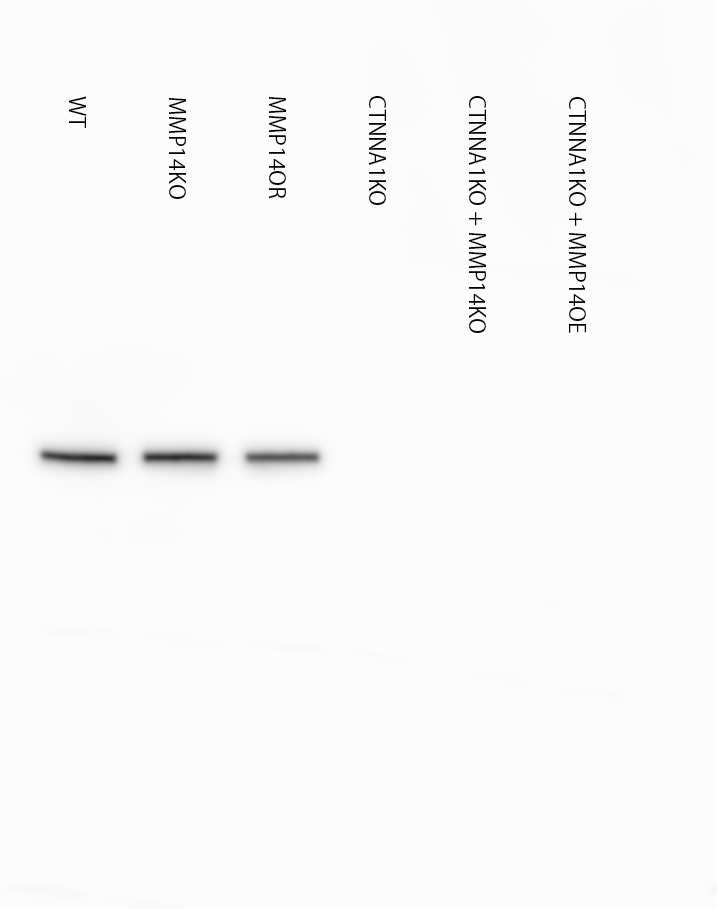

Supplement: Figure 3—figure supplement 1—source data 2. [file elife-76520-fig3-figsupp1-data2.zip › Figure 3 - figure supplement 1 - source data2/Figure 3 - figure supplement 1f Blot of alpha catenin with label.png]

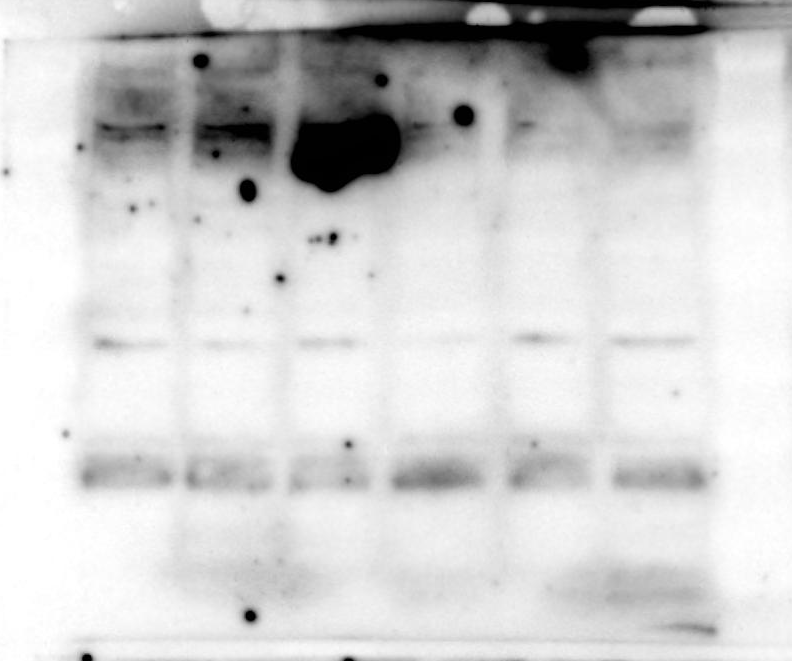

Supplement: Figure 3—figure supplement 1—source data 2. [file elife-76520-fig3-figsupp1-data2.zip › Figure 3 - figure supplement 1 - source data2/Figure 3 - figure supplement 1f Blot of Vimentin.png]

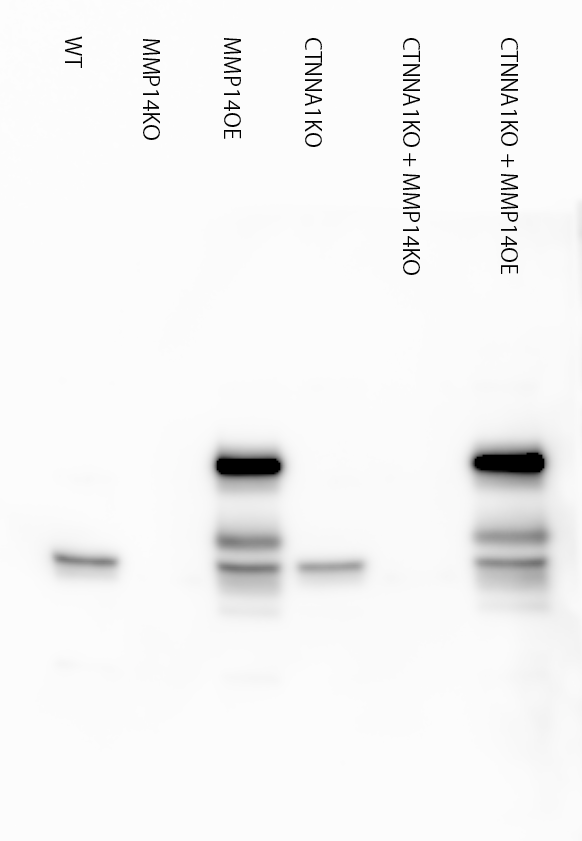

Supplement: Figure 3—figure supplement 1—source data 2. [file elife-76520-fig3-figsupp1-data2.zip › Figure 3 - figure supplement 1 - source data2/Figure 3 - figure supplement 1f Blot of MMP14 with label.png]

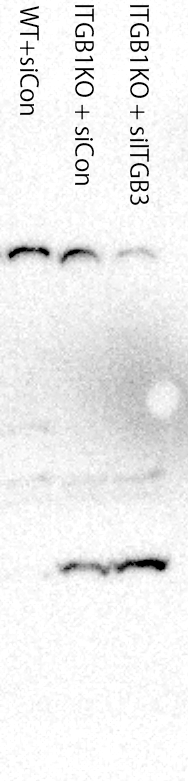

Supplement: Figure 4—figure supplement 1—source data 2. [file elife-76520-fig4-figsupp1-data2.zip › Figure 4 - figure supplement 1 - source data2/Figure 4 - figure supplement 1d Blot of integrin beta 3 with label.png]

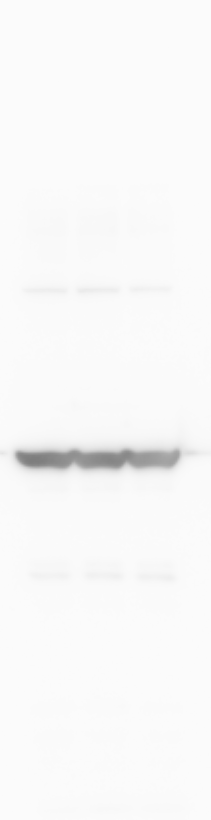

Supplement: Figure 4—figure supplement 1—source data 2. [file elife-76520-fig4-figsupp1-data2.zip › Figure 4 - figure supplement 1 - source data2/Figure 4 - figure supplement 1d Blot of beta actin.png]

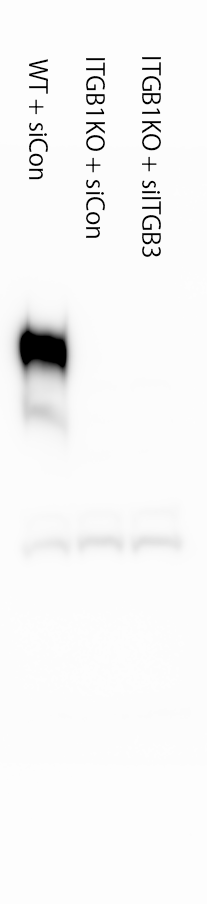

Supplement: Figure 4—figure supplement 1—source data 2. [file elife-76520-fig4-figsupp1-data2.zip › Figure 4 - figure supplement 1 - source data2/Figure 4 - figure supplement 1d Blot of integrin beta 1 with label.png]

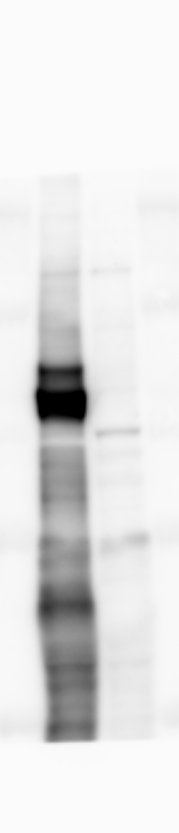

Supplement: Figure 4—figure supplement 1—source data 2. [file elife-76520-fig4-figsupp1-data2.zip › Figure 4 - figure supplement 1 - source data2/Figure 4 - figure supplement 1b Blot of integrin beta 1.png]

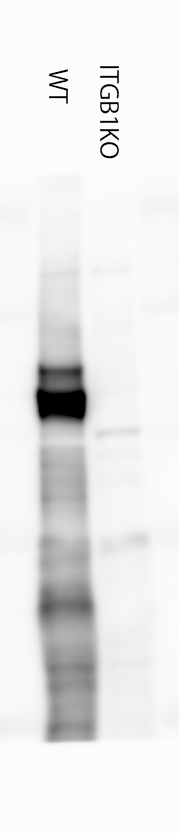

Supplement: Figure 4—figure supplement 1—source data 2. [file elife-76520-fig4-figsupp1-data2.zip › Figure 4 - figure supplement 1 - source data2/Figure 4 - figure supplement 1b Blot of integrin beta 1 with label.png]

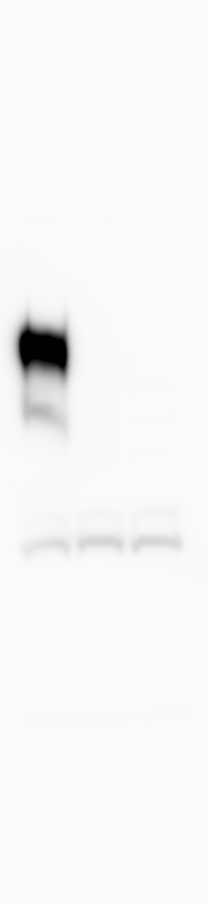

Supplement: Figure 4—figure supplement 1—source data 2. [file elife-76520-fig4-figsupp1-data2.zip › Figure 4 - figure supplement 1 - source data2/Figure 4 - figure supplement 1d Blot of integrin beta 1.png]

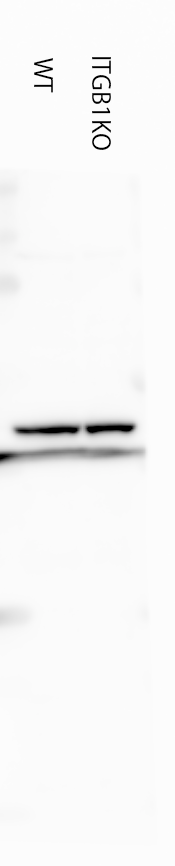

Supplement: Figure 4—figure supplement 1—source data 2. [file elife-76520-fig4-figsupp1-data2.zip › Figure 4 - figure supplement 1 - source data2/Figure 4 - figure supplement 1b Blot of beta actin with label.png]

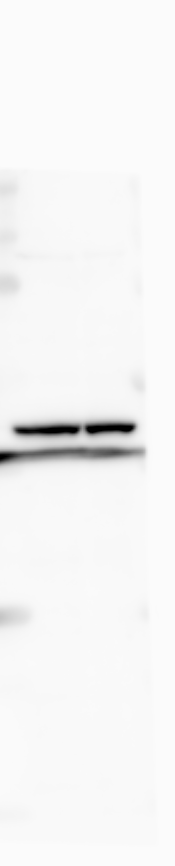

Supplement: Figure 4—figure supplement 1—source data 2. [file elife-76520-fig4-figsupp1-data2.zip › Figure 4 - figure supplement 1 - source data2/Figure 4 - figure supplement 1b Blot of beta actin.png]

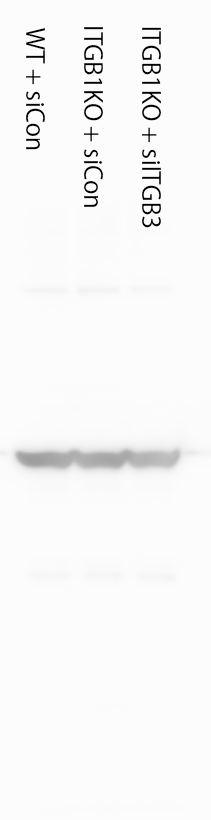

Supplement: Figure 4—figure supplement 1—source data 2. [file elife-76520-fig4-figsupp1-data2.zip › Figure 4 - figure supplement 1 - source data2/Figure 4 - figure supplement 1d Blot of beta actin with label.png]
