# Supplementary material for: Interplay of adherens junctions and matrix proteolysis determines the invasive pattern and growth of squamous cell carcinoma
Source: eLife. 2023 Mar 9;12:e76520. doi: 10.7554/eLife.76520 (PMC9998089; doi:10.7554/eLife.76520)
Supplement: Transparent reporting form [file elife-76520-transrepform1.docx]

***eLife’s* transparent reporting form**

We encourage authors to provide detailed information *within their submission* to facilitate the interpretation and replication of experiments. Authors can upload supporting documentation to indicate the use of appropriate reporting guidelines for health-related research (see [EQUATOR](http://www.equator-network.org/) [Network](http://www.equator-network.org/)), life science research (see the [BioSharing Information Resource](https://biosharing.org/)), or the ARRIVE guidelines for reporting work involving animal research. Where applicable, authors should refer to any relevant reporting standards documents in this form.

If you have any questions, please consult our Journal Policies and/or contact us: [editorial@elifesciences.org.](mailto:editorial@elifesciences.org)

# Sample-size estimation

- You should state whether an appropriate sample size was computed when the study was being designed
- You should state the statistical method of sample size computation and any required assumptions
- If no explicit power analysis was used, you should describe how you decided what sample (replicate) size (number) to use

Please outline where this information can be found within the submission (e.g., sections or figure

legends), or explain why this information doesn’t apply to your submission:

For the experimental work, we were cognisant of the time and resource implications of performing large numbers of replicates. Therefore, we followed the almost universal practice of performing a minimum of three replicates.

For simulated data, variance for each metric was estimated from trial simulations and the sample-size set to balance computation time and significance at a desired effect-size for all metrics. For example, we determined that an effect-size of 0.5 was reasonable for number of neighbours. Estimating the variance to be 0.1 and with a significance level of 0.05 and a power level of 0.9 results in a required sample-size of 9. Similar reasoning for the other metrics led to a sample-size of ten being adopted for each parameter combination, resulting in thousands of simulations taking multiple weeks of computation.

# Replicates

- You should report how often each experiment was performed
- You should include a definition of biological versus technical replication
- The data obtained should be provided and sufficient information should be provided to indicate the number of independent biological and/or technical replicates
- If you encountered any outliers, you should describe how these were handled
- Criteria for exclusion/inclusion of data should be clearly stated
- High-throughput sequence data should be uploaded before submission, with a private link for reviewers provided (these are available from both GEO and ArrayExpress)

Please outline where this information can be found within the submission (e.g., sections or figure

legends), or explain why this information doesn’t apply to your submission:

Three to five biological replicates were performed for all experimental work. A biological replicate is defined as an experiment that is set up and conducted on a different day. The exact number of replicates is stated in the figure legends.

We did not exclude any outliers in the experimental data. In the computational work, we excluded four simulations in which the spheroid become ‘hyper fragmented’ and, therefore, our collective metrics were rendered meaningless. This is mentioned on page 19 lines 679-684.

# Statistical reporting

- Statistical analysis methods should be described and justified
- Raw data should be presented in figures whenever informative to do so (typically when N per group is less than 10)
- For each experiment, you should identify the statistical tests used, exact values of N, definitions of center, methods of multiple test correction, and dispersion and precision measures (e.g., mean, median, SD, SEM, confidence intervals; and, for the major substantive results, a measure of effect size (e.g., Pearson's r, Cohen's d)
- Report exact p-values wherever possible alongside the summary statistics and 95% confidence intervals. These should be reported for all key questions and not only when the p-value is less than 0.05.

Please outline where this information can be found within the submission (e.g., sections or figure

legends), or explain why this information doesn’t apply to your submission:

Information about the statistical tests used and the ‘N’ number is provided in the legends. 95% confidence are shown on the plots. The concise figure layout precludes reporting all values for all comparisons. This data is included in the source data files.

(For large datasets, or papers with a very large number of statistical tests, you may upload a single table file with tests, Ns, etc., with reference to sections in the manuscript.)

# Group allocation

- Indicate how samples were allocated into experimental groups (in the case of clinical studies, please specify allocation to treatment method); if randomization was used, please also state if restricted randomization was applied
- Indicate if masking was used during group allocation, data collection and/or data analysis

Please outline where this information can be found within the submission (e.g., sections or figure legends), or explain why this information doesn’t apply to your submission:

Group allocation is not relevant for the majority of work in this manuscript. In the case of experimental work using cell lines, the different manipulations were performed on the same starting cell line. As the great majority of the experimental work was performed by one researcher, Takuya Kato; therefore, masking was not feasible. In the case of the mouse work, mice were randomly assigned to be injected with different cells and the tumour size measurements were made by technical staff at the Crick with no knowledge of the hypothesis being tested.

# Additional data files (“source data”)

- We encourage you to upload relevant additional data files, such as numerical data that are represented as a graph in a figure, or as a summary table
- Where provided, these should be in the most useful format, and they can be uploaded as

“Source data” files linked to a main figure or table

- Include model definition files including the full list of parameters used
- Include code used for data analysis (e.g., R, MatLab)
- Avoid stating that data files are “available upon request”

Please indicate the figures or tables for which source data files have been provided:

CC3D code overview can be found in Appendix 1 and all source code at <https://github.com/RobertPJenkins/kato_jenkins_et_al_CC3D>. The walkthrough includes both comprehensive overview of all code and detailed descriptions of mathematical operations used. MATLAB code used to generate the metrics can be found at the same link. CC3D model parameters used can be found in Table 1 and Appendix 2. A description of methodology used to determine parameter values from experimental data can be found in Subsection 1.4 (pages 3-5) of Appendix 1.
